# Amelioration of CKD-induced cardiomyocyte hypertrophy by pegmolesatide: involvement of JAK2/STAT3 inhibition and mitochondrial protection

**DOI:** 10.3389/fphar.2026.1806374

**Published:** 2026-05-13

**Authors:** Xinyu Zhang, Sufang Li, Tingru Lin, Liangying Gan, Hanze Zheng, Li Zhu, Li Zuo

**Affiliations:** 1 Department of Nephrology, Peking University People’s Hospital, Beijing, China; 2 Department of Cardiology, Beijing Key Laboratory of Early Prediction and Intervention of Acute Myocardial Infarction, Center for Cardiovascular Translational Research, Peking University People’s Hospital, Beijing, China; 3 Department of Basic Medical Sciences, Peking University Health Science Center, Beijing, China

**Keywords:** cardiac hypertrophy, CKD, JAK2/STAT3 signaling pathway, mitochondrial dysfunction, pegmolesatide

## Abstract

**Introduction:**

Cardiovascular disease represents the leading cause of mortality among patients with chronic kidney disease (CKD). CKD-associated cardiomyopathy is characterized by pathological left ventricular hypertrophy and myocardial fibrosis. Pegmolesatide, a novel pegylated continuous erythropoietin receptor activator, has demonstrated efficacy in treating renal anemia. However, the biological effects of Pegmolesatide beyond erythropoiesis remain poorly characterized.

**Methods:**

This study established a 5/6 nephrectomy rat model of CKD. The animals were randomized into four groups: sham-operated (Sham), sham-operated plus Pegmolesatide (Sham + Pegmolesatide), CKD model (CKD), and CKD model plus Pegmolesatide (CKD + Pegmolesatide). Cardiac function was assessed by echocardiography, while histopathological examination evaluated cardiac and renal structural changes. Serum renal function parameters were measured using biochemical assays. Myocardial expression of proteins and mRNAs related to fibrosis, hypertrophy, and associated signaling pathways was analyzed by Western blot and quantitative PCR. Furthermore, transcriptomic sequencing of myocardial tissue was performed, and in vitro experiments were conducted using AC16 human cardiomyocytes stimulated with serum from stage 5 CKD patients.

**Results:**

Transcriptomic sequencing suggested that the cardioprotective effects of Pegmolesatide might be associated with improved mitochondrial function and modulation of the STAT3 signaling pathway. Results demonstrated that Pegmolesatide significantly improved cardiac functional parameters and attenuated both myocardial fibrosis and cardiomyocyte hypertrophy. Mechanistic investigations revealed that these cardioprotective effects were mediated through inhibition of JAK2/STAT3 signaling pathway activation and concomitant improvement of mitochondrial function.

**Discussion:**

These findings demonstrate that pegmolesatide exerts anti-anemic effect-independent cardioprotection, offering novel insights into treating CKD-associated cardiomyopathy.

## 1 Introduction

Chronic kidney disease (CKD), affecting approximately 13.4% of the global population, represents a major public health challenge (Eckardt et al., 2013; Remuzzi et al., 2016). Its progression to end-stage renal disease (ESRD) is accelerating, with a projected 50%–100% increase in patients requiring kidney replacement therapy by 2030 (Jankowski et al., 2021). Cardiovascular disease is the leading cause of mortality in this population, driven significantly by uremic cardiomyopathy (UCM). UCM is characterized by pathological myocardial fibrosis and hypertrophy, leading to left ventricular hypertrophy (LVH) and heart failure in 40%–60% of ESRD patients, which severely worsens prognosis (Maqbool et al., 2023; Valbuena-López et al., 2023). The severity of UCM strongly correlates with CKD progression, highlighting the need for early cardiac monitoring and intervention (Ronco et al., 2008).

The pathophysiology of UCM is centrally driven by the accumulation of uremic toxins (Song et al., 2025). Protein-bound toxins, such as indoxyl sulfate (IS) and p-cresyl sulfate (PCS), cross the endothelium to directly impair cardiomyocytes (Lekawanvijit, 2018). Their mechanisms include inducing mitochondrial dysfunction, oxidative stress, and disrupted calcium homeostasis, thereby compromising contractility (Yisireyili et al., 2013; Taylor et al., 2015; Thome et al., 2020). Furthermore, toxins like IS activate pro-hypertrophic and pro-fibrotic pathways, including MAPK and NF-κB signaling, within a milieu of sustained inflammation and oxidative stress, creating a vicious cycle that promotes maladaptive remodeling and heart failure (Lekawanvijit and Krum, 2015; Wang and Shapiro, 2019; Yang J. et al., 2022). Therefore, counteracting these toxins or their downstream effects is a promising therapeutic avenue for UCM.

Mitochondrial dysfunction has been demonstrated to play a pivotal role in the progression of uremic cardiomyopathy (UCM). In patients with uremia, mitochondria within the affected heart muscle demonstrate impaired bioenergetics, which can be characterized by the presence of chronic respiratory uncoupling, a reduced respiratory control ratio, and compromised oxidative phosphorylation efficiency. This leads to a reduced phosphocreatine/ATP ratio, depleting myocardial energy reserves (Taylor et al., 2015). Concurrently, mitochondria become a major source of reactive oxygen species (ROS) through complexes I/III, NOX4, and p66Shc signaling, exacerbating oxidative stress. This redox imbalance sensitizes the mitochondrial permeability transition pore (mPTP), promoting its opening and accelerating cardiomyocyte loss *via* apoptosis and necrosis (Peoples et al., 2019). Further dysfunction arises from uremia-induced calcium dysregulation, involving altered handling *via* the mitochondrial Na^+^/Ca^2+^ exchanger (NCLX), which impairs ATP synthesis. Impaired insulin signaling also reduces metabolic flexibility and increases susceptibility to injury (Patel et al., 2022). Collectively, these interconnected mechanisms establish mitochondrial failure as a pivotal therapeutic target in UCM. Promising strategies include mitochondria-targeted antioxidants, SGLT2 inhibitors, and L-carnitine, which have shown potential in preclinical models to improve mitochondrial function and cardiac outcomes.

Pegmolesatide is a novel synthetic, PEGylated erythropoietin-mimetic peptide that exerts its long-acting erythropoietic effect by uniquely and stably binding to and activating the erythropoietin receptor, and is indicated for the treatment of anemia associated with chronic kidney disease (CKD) (Ma et al., 2025). The medication’s primary benefit is its once-monthly administration schedule, which has been demonstrated to enhance patient compliance. A multitude of clinical studies have demonstrated the non-inferior efficacy of the subject in question, and in some aspects, its superiority to conventional Erythropoiesis-Stimulating Agents (ESAs), requiring more frequent dosing. Regarding cardiovascular safety, Phase III clinical trials provided compelling data: in dialysis-dependent patients, the incidence of composite cardiovascular events (including all-cause mortality, stroke, and myocardial infarction) was 2.4% in the Pegmolesatide group compared to 4.0% in the epoetin alfa group, with a hazard ratio (HR) of 0.47 (95% CI: 0.14–1.59). Furthermore, the incidence of other cardiovascular events (e.g., hospitalization for heart failure) was 1.2% in the Pegmolesatide group, lower than the 4.0% observed in the control group (HR 0.28; 95% CI: 0.07–1.17) (Zhang et al., 2023). Similarly, in non-dialysis-dependent patients, Pegmolesatide demonstrated a favorable profile, with a composite safety event incidence of 0.9% versus 3.4% in the epoetin alfa group (HR 0.26; 95% CI: 0.02–3.03). Notably, no hospitalizations for heart failure occurred in the Pegmolesatide group, whereas the incidence was 5.2% in the comparator group (Xie et al., 2025). Collectively, these clinical data indicate a favorable cardiovascular safety profile for Pegmolesatide in high-risk CKD patients, yet the mechanistic basis for its potential cardioprotective effects remains incompletely defined.

To this end, a comprehensive experimental strategy was employed to investigate the cardioprotective potential of pegmolesatide. This strategy included the stimulation of AC16 human cardiomyocytes with serum from uremic patients and evaluation in a well-established rat model of CKD (5/6 nephrectomy). The objective of this investigation is to elucidate the fundamental mechanisms underlying CKD-associated cardiomyopathy and to provide innovative therapeutic strategies for its management.

## 2 Materials and methods

### 2.1 Animals and CKD model establishment

All animal procedures were approved by the Peking University People’s Hospital Animal Care and Use Committee and complied with national animal welfare guidelines. Male Sprague-Dawley rats (8 weeks old) were maintained under standard conditions (12-h light/dark cycle, 22 °C) with free access to food and water. Chronic kidney disease (CKD) was induced *via* a two-stage 5/6 nephrectomy (5/6 Nx). Briefly, under anesthesia, approximately two-thirds of the left kidney was infarcted by ligating branches of the renal artery. One week later, the right kidney was surgically excised. Sham-operated rats underwent identical surgical procedures without kidney removal**,** specifically without undergoing left renal artery branch ligation or right nephrectomy, and only receiving separation of the renal capsule. All animals were allowed to recover for 3 weeks after the final surgery before treatment initiation.

### 2.2 Experimental groups and drug administration

**FIGURE 1:**
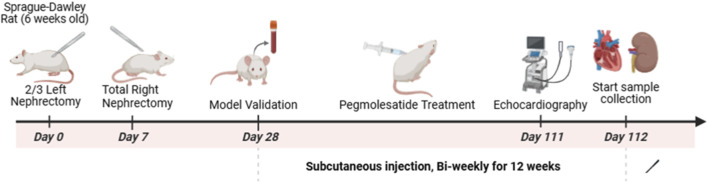
Schematic of the animal model.

Rats were randomly allocated to four experimental groups (n = 6 per group): Sham group (Sham-operated rats receiving vehicle),Sham + Pegmolesatide group (Sham-operated rats treated with Pegmolesatide), 5/6 Nx group (CKD model rats receiving vehicle) and 5/6 Nx + Pegmolesatide group (CKD model rats treated with Pegmolesatide). Pegmolesatide was administered subcutaneously at a dose of 0.1 mg/kg on a biweekly basis. The selection of this dosing regimen was based on allometric scaling using body surface area. The clinical human dose of pegmolesatide (0.04 mg/kg once monthly) was converted to an equivalent rat dose using a standard human-to-rat conversion factor of approximately 5.6, yielding 0.224 mg/kg/month. This was divided into two biweekly subcutaneous injections of 0.112 mg/kg, rounded to 0.1 mg/kg per injection. Treatment was initiated 3 weeks after the final surgical procedure (to allow for CKD development) and continued for a total of 12 weeks. Rats in the vehicle-control groups received an equivalent volume of saline on the same schedule. Body weight was meticulously monitored on a weekly basis throughout the entirety of the study period, commencing from the period preceding the surgical procedure and continuing until the point of tissue harvest at the conclusion of the study. Serum creatinine, blood urea nitrogen (BUN), and complete blood counts were assessed at two time points: 3 weeks after surgery (prior to treatment initiation) and before the terminal tissue collection at the study endpoint. At the terminal stage, 24-h urine samples were collected for the measurement of urinary creatinine and protein excretion. The urinary protein concentration was determined using the Coomassie Brilliant Blue (CBB) method, and the urinary creatinine concentration was measured using a commercially available creatinine assay kit (Jiancheng Bioengineering Institute, Nanjing, China) (Figure 1).

### 2.3 Echocardiography

Cardiac function was evaluated at the end of the 12-week treatment period using a high-resolution ultrasound imaging system (Vevo FS, VisualSonics). The rats were rendered mildly sedated using isoflurane (1.5%–2%), which was administered via a nasal cannula. The echocardiographic measurements (N = 6 per group) encompassed left ventricular internal dimensions (LVIDd, LVIDs), wall thicknesses (IVSd, IVSs, LVAWd, LVAWs, LVPWd, LVPWs), ejection fraction (EF), and fractional shortening (FS). The left ventricular mass (LVM) was then calculated.

### 2.4 RNA isolation and RNA sequencing

Left ventricular apex tissues were collected from rats that had undergone either sham operation or 5/6 nephrectomy (5/6 Nx), with or without pegmolesatide treatment for a period of 12 weeks. Three biological replicates per group were used for RNA-seq. Total RNA was extracted from these samples using TRIzol reagent (Invitrogen) according to the manufacturer’s instructions. The assessment of RNA concentration and purity was conducted using a Nanodrop 2000 spectrophotometer (Thermo Fisher Scientific). RNA integrity was assessed using an Agilent 2,100 Bioanalyzer (RIN ≥7.0). High-quality RNA libraries were constructed and sequenced on an Illumina NovaSeq 6,000 platform by Oebiotech Co., Ltd. (Shanghai, China). The raw sequencing data have been submitted to the NCBI Sequence Read Archive (SRA) under the BioProject accession number PRJNA1453710. The data will be publicly accessible at https://www.ncbi.nlm.nih.gov/sra/PRJNA1453710 following the release date specified in the submission.

### 2.5 Data collection and differentially expressed gene analysis

Initially, raw sequencing reads were subjected to a series of preprocessing steps to ensure the integrity of the data. Adapter and low-quality sequence contamination was subsequently removed using the fastp (Version 0.20.1) with the length required 50 parameters, thereby generating a set of high-quality, uncontaminated reads. Quality control (QC) metrics, including per-base sequence quality (Q20, Q30), GC content, and duplication rates, were assessed using FastQC (version 0.11.9). Subsequent alignment-based quality control of RNA-seq data was performed using RSeQC (version 4.0.0) with default parameters. To address the potential for ribosomal RNA contamination, the clean reads were aligned to the rat ribosomal RNA database (SILVA version 138) using Bowtie2 (version 2.4.2) with the “--very-sensitive” parameter. Reads that were mapped to ribosomal RNA (rRNA) were discarded, and the remaining unmapped reads were retained for subsequent analysis. Subsequently, these high-quality non-rRNA reads were aligned to the rat reference genome (Rn6_6.0/GCF_000001895.5, Ensembl release 104) using HISAT2(version 2.1.0) with the RNA-strandness RF parameters. The assembly and quantification of transcripts was performed using StringTie (version 2.1.4), and gene-level read counts were obtained with HTSeq-count (version 0.11.2) with the -s reverse parameter, SAM/BAM file processing and analysis were performed using Samtools (version 1.9). Subsequently, differential expression analysis was conducted between the experimental groups using DESeq2 (version 1.34.1). Genes with an adjusted p-value less than 0.05 and a |log2 (fold change) | greater than one were considered to be significantly differentially expressed.

### 2.6 Pathway enrichment analysis

The Kyoto Encyclopedia of Genes and Genomes (KEGG) pathway enrichment analysis was conducted on the sets of differentially expressed genes using the hypergeometric test implemented in clusterProfiler (version 4.0.0). This approach identifies KEGG pathways that are significantly overrepresented among the differentially expressed genes relative to the whole genome background. Gene Set Enrichment Analysis (GSEA) was performed using the fgsea package (version 1.18.0) with the MSigDB hallmark gene sets and GO gene sets. A nominal p-value <0.05 and false discovery rate (FDR) < 0.25 were considered statistically significant. The chord diagram linking DEGs to enriched pathways was generated using the GOplot package (version 1.0.2).

### 2.7 Histopathological and immunofluorescence analysis

Heart and kidney tissues were harvested and embedded in paraffin prior to being sectioned at a thickness of 4 μm. Cardiac sections were subjected to a multistep staining procedure, beginning with hematoxylin and eosin (H&E) to assess morphology, followed by Masson’s trichrome staining to visualize collagen, and Sirius red staining to evaluate fibrosis. WGA fluorescent staining was employed to delineate cardiomyocyte borders. Renal sections were subjected to hematoxylin and eosin (H&E) staining for structural observation, Masson’s trichrome staining for collagen deposition evaluation, and periodic acid Schiff (PAS) staining for glycogen and glycoprotein detection. All staining procedures were performed in accordance with standard protocols. Following the processing stage, the sections were subjected to thorough examination and imaging using both light and fluorescence microscopy.

### 2.8 Western blot analysis

Following the removal of heart tissue from −80 °C storage, frozen samples were homogenized in ice-cold RIPA lysis buffer (Beyotime, P0013 B) supplemented with protease and phosphatase inhibitors. The protein concentration was determined using a BCA assay kit (Thermo Fisher, 23,225). Subsequently, 30 μg of protein per sample was mixed with loading buffer, denatured, and separated by SDS-PAGE (Biorad, 4,569,033) under reducing conditions. Subsequent to electrophoresis, proteins were transferred onto PVDF membranes (Millipore, IPVH00010) employing a standard wet transfer system. The membranes were then blocked with 5% non-fat milk in TBST for 1 h at room temperature. This was followed by incubation overnight at 4 °C with the following primary antibodies: anti-STAT3 (Cell Signaling Technology, 9,139; 1:1,000), anti-phospho-STAT3 (Tyr705) (Cell Signaling Technology, 9,145; 1:1,000), anti-BNP (Abcam, ab19645; 1:800), anti-β-MHC (Santa Cruz, sc-53089; 1:500), and anti-GAPDH (Proteintech, 60004-1-Ig; 1:5,000) as a loading control. Subsequent to three washes with TBST, the membranes were incubated with the appropriate HRP-conjugated secondary antibodies for 1 hour at room temperature. The visualization of protein bands was accomplished through the utilization of an enhanced chemiluminescence substrate (ECL) detection kit (Millipore, WBKLS0500). Quantitative analysis of band intensity was performed using ImageJ software.

### 2.9 Quantitative real-time PCR (qPCR)

Total RNA was isolated from approximately 30 mg of pulverized heart tissue using TRIzol Reagent (Invitrogen). The RNA concentration and purity were measured using a NanoDrop spectrophotometer, with the A260/280 and A260/230 ratios being determined. 1 μg of total RNA was reverse transcribed into cDNA using a High-Capacity cDNA Reverse Transcription Kit (Takara, RR047 A). Quantitative polymerase chain reaction (qPCR) was performed in triplicate with PowerUp SYBR Green Master Mix (Takara, RR820 A) on a CFX96 Touch system (Bio-Rad). Each 20 µL reaction mixture contained 1× Master Mix, 0.2 µM of each primer, and 50 ng of cDNA. Subsequently, the expression levels of ANP, BNP, and β-MHC mRNA were subjected to analysis utilizing the 2^(-ΔΔCt)^ method. GAPDH was employed as the endogenous control.

### 2.10 Serum and hematological analysis

In order to assess renal function and systemic hematological changes, serum creatinine (Scr) and blood urea nitrogen (BUN) levels were quantified using standard biochemical kits. Furthermore, the concentration of hemoglobin (Hb) was measured to evaluate alterations in red blood cell status. All procedures were carried out in strict accordance with the protocols stipulated by the manufacturers.

### 2.11 Cell culture experiment

AC16 human cardiomyocytes were cultivated in DMEM-F12 medium. Subsequent to a 24-h plating period, cells were subjected to a 24-h serum starvation process using serum-free medium. The medium was then substituted with fresh DMEM-F12, which had been supplemented with 10% serum pooled from either healthy donors or patients with stage 5 chronic kidney disease (CKD). Baseline characteristics of healthy controls (n = 34) and stage 5 CKD patients (n = 35) are shown in Supplementary Table S1. Serum samples from CKD patients were pooled, filtered (0.22 μm), aliquoted, and stored at −80 °C, then diluted to 10% for experiments. The cells were then cultured for an additional 48 h. During this treatment period, cells were concurrently exposed to Pegmolesatide at a concentration of 1,000 ng/mL. The concentration was selected based on CCK-8 assay results, which aligned with effective doses reported in previous *in vitro* studies and were further supported by preliminary experimental data (Xie et al., 2025). The extraction of RNA occurred at 24 h post-induction in order to assess the mRNA expression of ANP, BNP, α-SMA, and collagen I. The collection of protein occurred at 48 h post-induction for the purpose of Western blot detection of β-MHC and BNP.

### 2.12 Measurement of mitochondrial reactive oxygen species (ROS)

The levels of mitochondrial superoxide were determined using a commercial probe, MitoSOX™ Red Mitochondrial Superoxide Indicator (from Thermo Fisher Scientific). Cells were cultivated on glass-bottom dishes or in multi-well plates until reaching 70%–80% confluence. Following treatment, cells were washed with warm phosphate-buffered saline (PBS) and incubated with 5 µM MitoSOX™ Red reagent in Hank’s Balanced Salt Solution (HBSS) at 37 °C for 30 min in the dark. Subsequent to the incubation period, the cells were washed thrice with warm HBSS in order to eliminate excess probe. Fluorescence imaging was performed using a confocal microscope with excitation/emission set at 390/610 nm. For the purpose of quantitative analysis, the intensity of the emitted fluorescence was measured using a microplate reader, with the measurement conducted at the same wavelengths. The data were then normalized to total protein content or cell number, and the results were expressed as fold change relative to the control group.

### 2.13 Lipid peroxidation assay

The extent of lipid peroxidation was determined by employing the BDPY581/591C11 fluorescent probe, which undergoes a shift in emission from red to green upon oxidation. A 2 µM working solution was prepared by diluting BDPY581/591 C11 (2 µM) in phosphate-buffered saline (PBS) at a 1:1,000 ratio. This solution was prepared under conditions that ensured protection from light. Following this, the culture medium was replaced with the staining working solution, and the solution was subsequently incubated at 37 °C for 20 min. Following a thorough cleansing with Phosphate Buffered Saline (PBS), a microscopic analysis of the cells was conducted. The suspension cells were initially stained in solution, subsequently washed, and then resuspended in phosphate-buffered saline (PBS) for analysis. Fluorescence was detected at 581/591 nm (reduced form) and 488/510 nm (oxidized form) using filter sets appropriate for Cy3/PI and GFP/FITC, respectively. The red-to-green fluorescence ratio was employed as a quantitative metric to assess lipid peroxidation.

### 2.14 Statistical analysis

The data are expressed as the mean ± standard deviation (SD). All statistical analyses were performed using GraphPad Prism software. For *in vivo* experiments, n = 6 per group, representing individual animals. For *in vitro* experiments, n = 3 per group, representing independent biological replicates. For the purpose of conducting a comparison between two groups, an unpaired two-tailed Student’s t-test was employed. Comparisons across multiple groups were analyzed by one-way or two-way ANOVA, as appropriate, followed by Bonferroni’s post hoc test for multiple comparisons. A p-value of less than 0.05 was considered statistically significant.

## 3 Results

### 3.1 Pegmolesatide Ameliorates Structural but Not Functional Damage in CKD

**FIGURE 2:**
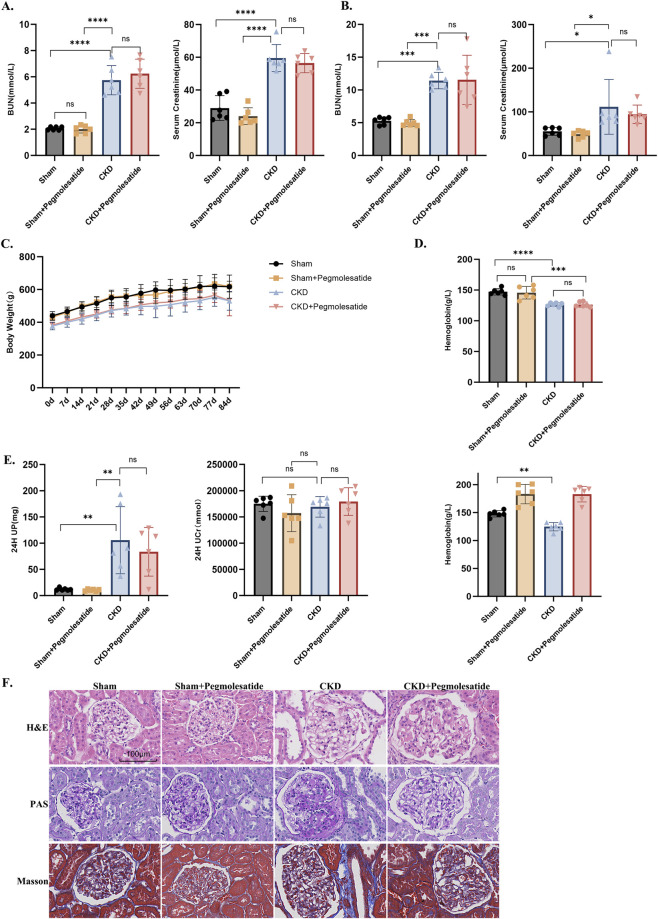
Pegmolesatide Ameliorates Structural but Not Functional Damage in CKD. **(A)** Serum levels of blood urea nitrogen (BUN) and creatinine in rats at 3 weeks after surgery (before drug administration). **(B)** Serum BUN and creatinine levels at terminal blood collection after 12 weeks of drug administration. **(C)** Body weight monitoring in rats following the first dose and throughout the experimental period until sacrifice. **(D)** Hemoglobin levels were monitored, with data from before administration and at the terminal time point included for statistical analysis. **(E)** Quantification of 24-h urinary protein and urinary creatinine from terminal urine collection. **(F)** Representative micrographs of kidney tissue sections stained with Hematoxylin and Eosin (HE, for histomorphology), Periodic Acid-Schiff (PAS, for glycogen and basement membranes), and Masson’s trichrome (for collagen fiber deposition), illustrating renal pathological injury and repair, scale bar = 100 μm (n = 6, Data are presented as mean ± SD, *p < 0.05, **p < 0.01, ***p < 0.001, ****p < 0.0001 vs. Sham & Sham + Pegmolesatide group, ##*p* < 0.01 vs. CKD group; ns, not significant).

The efficacy of the chronic kidney disease (CKD) model was confirmed by an assessment of serum creatinine (Scr) and blood urea nitrogen (BUN) levels at both 3 weeks after 5/6 nephrectomy (5/6 Nx) and at the study endpoint. These levels were significantly elevated compared with the Sham group (Figures 2A,B). During the 12-week period of drug administration, weekly body weight monitoring revealed no significant differences between the Pegmolesatide-treated group and the untreated CKD model group (Figure 2C). Furthermore, the levels of hemoglobin were measured at two time points: 3 weeks after the surgical procedure and at the final blood sampling. A comparison of the CKD rats with the sham-operated group revealed a substantial decrease in hemoglobin levels (from 147.8 g/L in the sham group to 124.7 g/L in the CKD model group). Notably, this decrease was restored following Pegmolesatide treatment (Figure 2D). At the conclusion of the treatment period, 24-h urine collection prior to sample harvesting revealed a significant increase in urinary creatinine and protein levels in the 5/6 Nx group. However, the administration of Pegmolesatide did not result in a substantial alteration of these renal function parameters (Figure 2E). A histopathological evaluation of kidney tissues, employing hematoxylin and eosin (H&E), periodic acid–Schiff (PAS), and Masson’s trichrome staining techniques, revealed severe interstitial fibrosis and substantial renal tubular injury in the CKD group. Conversely, Pegmolesatide treatment exhibited a partial amelioration of these pathological changes (Figure 2F). These findings suggest that, despite its apparent lack of significant impact on renal function parameters or body weight within the experimental parameters established, Pegmolesatide offers a degree of protection against renal structural injury.

### 3.2 Pegmolesatide improves cardiac function in CKD rats

**FIGURE 3:**
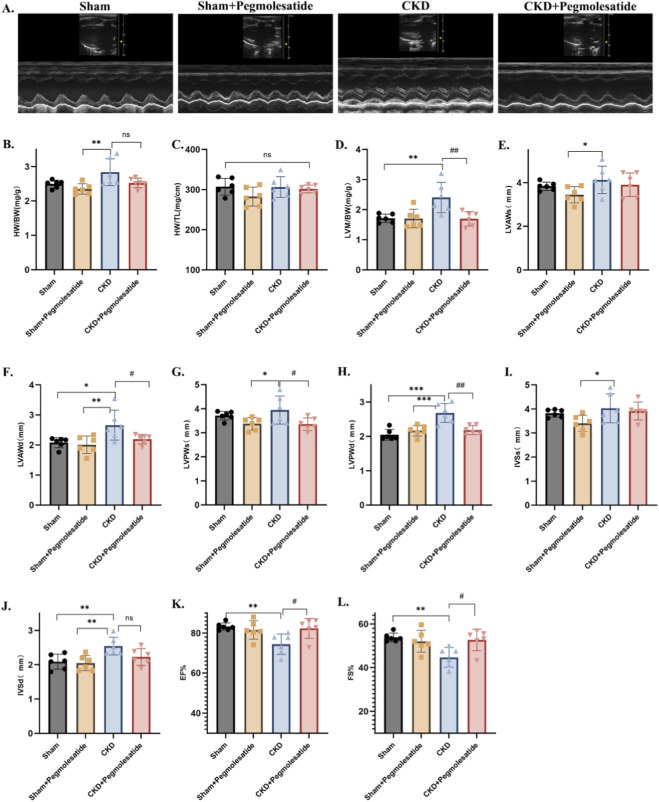
Effects of Pegmolesatide on cardiac structure and function in CKD Rats. **(A)** Representative M-mode echocardiographic images of the heart from each group **(B–L)** Echocardiographic parameters of cardiac function and structure: **(B)** Heart weight to body weight ratio (HW/BW). **(C)** Heart weight to tibia length ratio (HW/TL). **(D)** Left ventricular weight to body weight ratio (LVW/BW). **(E)** Left ventricular anterior wall thickness at end-systole (LVAWs). **(F)** Left ventricular anterior wall thickness at end-diastole (LVAWd). **(G)** Left ventricular posterior wall thickness at end-systole (LVPWs). **(H)** Left ventricular posterior wall thickness at end-diastole (LVPWd). **(I)** Interventricular septal thickness at end-systole (IVSs). **(J)** Interventricular septal thickness at end-diastole (IVSd). **(K)** Left ventricular ejection fraction (LVEF) **(L)** Left ventricular fractional shortening (LVFS) (Data are presented as mean ± SD. *p < 0.05, **p < 0.01, ***p < 0.001 vs. Sham group; #*p* < 0.05, ##*p* < 0.01 vs. CKD group; ns, not significant).

A comprehensive echocardiographic evaluation was conducted to assess cardiac structure and function. Representative M-mode images from each group are displayed in Figure 3A. At the conclusion of the experimental period, no significant differences were observed in either the heart weight/body weight ratio or the heart weight/tibia length ratio among the groups (Figures 3B,C). However, the left ventricular weight/body weight ratio was significantly higher in CKD rats compared with the sham and Pegmolesatide-treated groups (Figure 3D). Furthermore, both the left ventricular anterior wall diastolic thickness (LVAWd) and the posterior wall diastolic thickness (LVPWd) were markedly increased in the CKD group relative to the sham and treatment groups, whereas no significant difference in interventricular septal diastolic thickness (IVSd) was detected between the CKD and Pegmolesatide-treated groups (Figures 3E–J). In regard to cardiac function, rats subjected to 5/6 nephrectomy (5/6 Nx) exhibited significant impairment, characterized by a notable reduction in left ventricular systolic function—specifically, in ejection fraction (EF) and fractional shortening (FS), though these values did not reach the threshold for heart failure. Treatment with Pegmolesatide led to a significant amelioration of these abnormalities, effectively restoring systolic function parameters (EF and FS) and attenuating pathological ventricular remodeling (Figures 3K,L). Collectively, these findings demonstrate that Pegmolesatide effectively mitigates CKD-induced cardiac hypertrophy, improves systolic function, and slows the progression of uremic cardiomyopathy in this model.

### 3.3 Pegmolesatide attenuates myocardial hypertrophy in CKD rats

**FIGURE 4:**
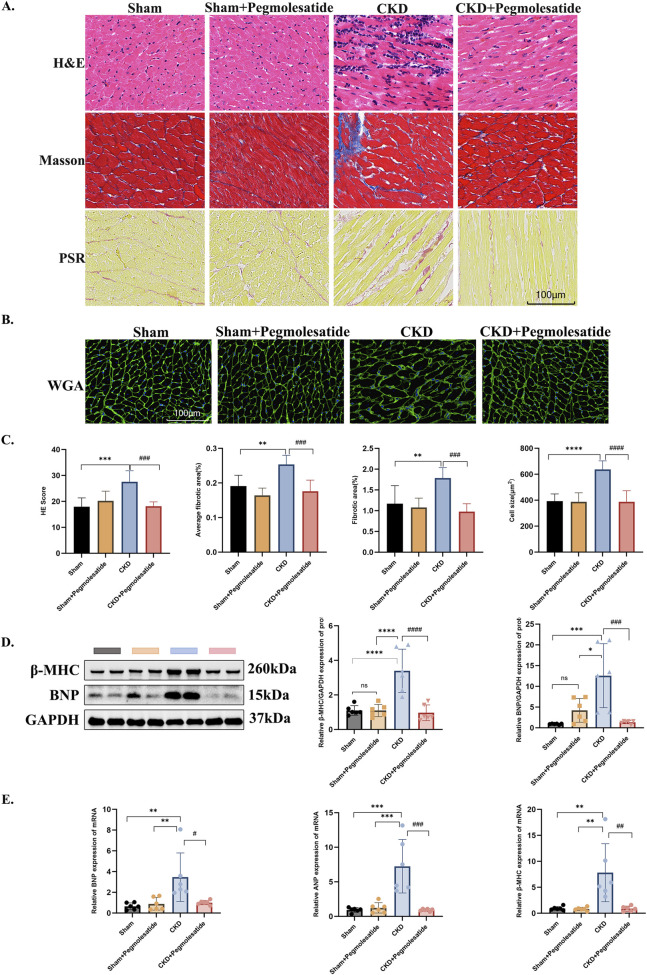
Effects of Pegmolesatide on myocardial histomorphology and the expression of hypertrophy markers in a CKD rat model. **(A)** Representative images of myocardial tissue staining: H&E staining (for general myocardial cytoarchitecture); Masson’s trichrome staining (myocardium in red, collagen fibers in blue); Picrosirius red (PSR) staining (collagen deposition appears red), scale bar = 100 μm. **(B)** Wheat germ agglutinin (WGA) fluorescent staining (green, scale bar = 100 μm, for visualizing cardiomyocyte cross-sectional area). **(C)** The statistical results of pathological staining including HE, Masson, Sirius Red and WGA. **(D)** Western blot analysis of myocardial tissue: Representative immunoblots for the myocardial hypertrophy markers beta-myosin heavy chain (β-MHC) and brain natriuretic peptide (BNP). **(E)** Quantitative real-time PCR (qPCR) analysis of myocardial mRNA expression levels for hypertrophy-related genes: atrial natriuretic peptide (ANP), BNP, and β-MHC (Data are presented as mean ± SD. *p < 0.05, **p < 0.01, ***p < 0.001 vs. Sham group; #p < 0.05, ##p < 0.01, ###p < 0.001, ####p < 0.0001 vs. CKD group; ns, not significant).

Histochemical analyses have yielded the following findings: Histological evaluation (HE staining) revealed that pegmolesatide significantly ameliorated the disarray of cardiomyocyte alignment and diminished inflammatory cell infiltration. Masson’s trichrome staining revealed a significant attenuation of aberrant accumulation of interstitial collagen fibers by pegmolesatide. Sirius red staining revealed the presence of red-stained collagen fibers in the myocardial interstitium of the model group. These fibers were significantly reduced by pegmolesatide, indicating a decrease in total collagen volume (Figure 4A). WGA staining revealed a substantial augmentation in cardiomyocyte cross-sectional area in the model group relative to the sham group. This augmentation was effectively mitigated by pegmolesatide treatment (Figure 4B). Figure 4C presents the quantitative analysis of HE, Masson, Sirius Red and WGA pathological staining images. At the molecular level, Western blot analysis revealed a significant increase in the protein expression levels of myocardial hypertrophy markers, including β-myosin heavy chain (β-MHC) and B-type natriuretic peptide (BNP), in the myocardial tissue of the CKD group compared to the sham group. Pegmolesatide treatment led to a reduction in the expression of these pathological hypertrophy markers (Figure 4D). These findings were further corroborated at the mRNA level by RT-PCR, which demonstrated that the transcript levels of BNP, ANP, and β-MHC in the pegmolesatide-treated groups were significantly lower than those in the CKD group (Figure 4E). The findings reveal that pegmolesatide exerts a robust inhibitory effect on pathological myocardial hypertrophy, manifesting in a consistent trend at both the protein and mRNA levels. This suppression is evident at both the transcriptional and translational levels, underscoring the comprehensive nature of the therapeutic intervention.

### 3.4 Pegmolesatide attenuates myocardial remodeling by modulating JAK/STAT signaling and mitochondrial function: Insights from transcriptomic profiling

**FIGURE 5:**
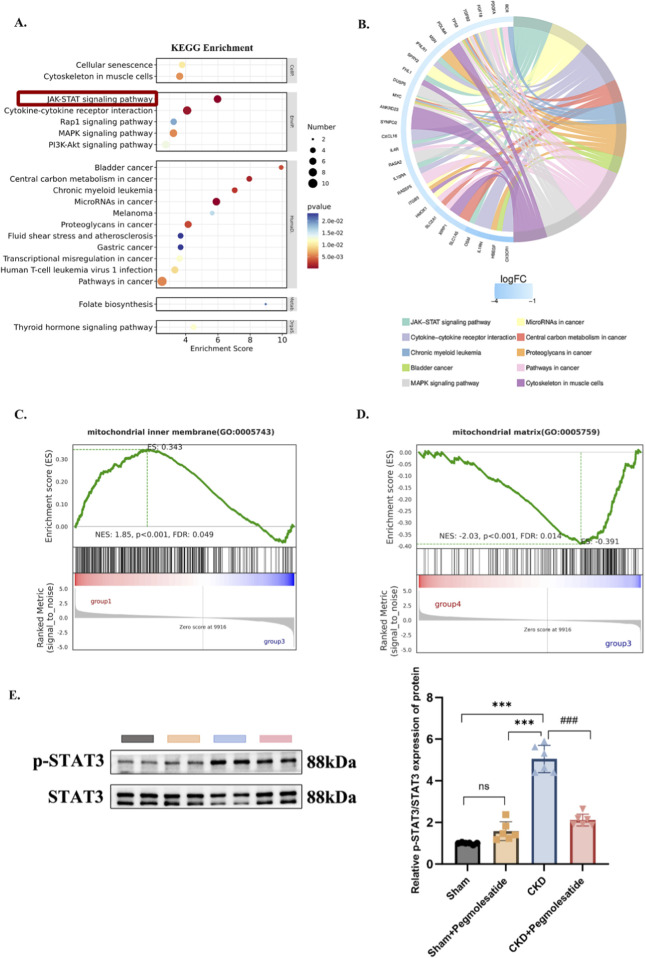
Modulation of JAK/STAT Signaling and Mitochondrial Function Mediates the Cardioprotective Effect of Pegmolesatide. **(A)** KEGG enrichment bubble chart: Pathways significantly enriched among differential genes are shown. Bubble size indicates the number of enriched genes, and color represents enrichment significance (darker color indicates higher significance). The JAK-STAT signaling pathway is markedly enriched (highlighted in red). **(B)** Enrichment chord diagram: Associations between differential genes and enriched pathways are displayed, with colored blocks representing different pathways and connecting lines indicating gene-pathway involvement. **(C,D)** GSEA plots: Enrichment results for the mitochondrial inner membrane gene set (GO:0005743) and mitochondrial matrix gene set (GO:0005759) are presented. **(E)** STAT3 phosphorylation detection in rat myocardial tissue Left panel shows Western blot bands for p-STAT3 and STAT3. Right panel displays relative expression levels (Data are presented as mean ± SD, n = 6, ***p < 0.001 vs. Sham or Sham + Pegmolesatide groups, ###*p* < 0.001 vs. CKD + Pegmolesatide groups).

A comprehensive investigation was conducted to elucidate the underlying mechanisms of Pegmolesatide-mediated cardioprotection. To this end, transcriptomic sequencing of myocardial tissue was performed. The differentially expressed genes (DEGs) between the CKD model group and the Sham control group were visualized in a volcano plot (Supplementary Figure S1). Subsequent functional enrichment analysis, incorporating both KEGG and Gene Set Enrichment Analysis (GSEA), identified the key pathways involved. KEGG analysis revealed a significant enrichment of the JAK-STAT signaling pathway (Supplementary Figure S2; Figure 5A). The association between differential genes and these enriched pathways is visually summarized in the chord diagram (Figure 5B). GSEA further confirmed the significant enrichment of both the mitochondrial matrix (GO:0005759) and, notably, gene sets related to mitochondrial inner membrane function (GO:0005743), as supported by the respective normalized enrichment score (NES), p-value, and false discovery rate (FDR) (Figures 5C,D). Western blot analysis provided confirmatory evidence at the protein level, demonstrating that the marked increase in STAT3 phosphorylation in the myocardial tissue of rats with chronic kidney disease (CKD) was substantially attenuated by Pegmolesatide treatment (Figure 5E), indicating a direct modulatory effect on this signaling cascade.

### 3.5 Pegmolesatide attenuates hypertrophic and fibrotic responses in AC16 cardiomyocyte model

**FIGURE 6:**
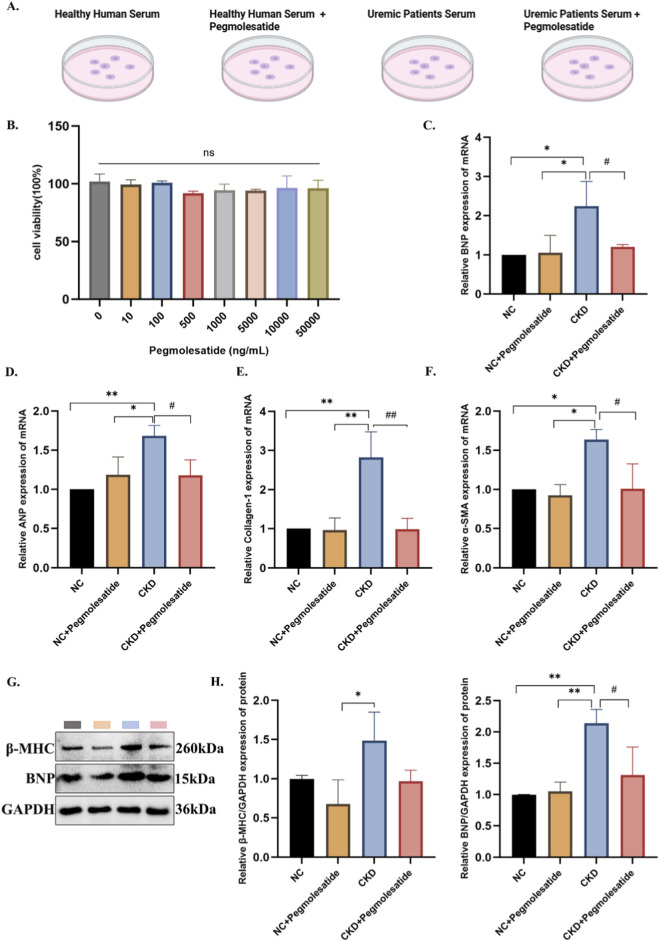
Pegmolesatide Attenuates Uremic Serum-Induced Hypertrophic and Fibrotic Responses in AC16 cells. **(A)** Schematic of cell treatment groups: healthy human serum, healthy human serum + Pegmolesatide, uremic serum, and uremic serum + Pegmolesatide. **(B)** Cell viability (CCK-8 assay) after treatment with Pegmolesatide (0–50,000 ng/mL). **(C–F)** Relative mRNA levels (qPCR) of **(C)** BNP **(D)** ANP, **(E)** Collagen-I, and **(F)** α-SMA. **(G,H)** Relative protein expression of β-MHC and BNP (*p < 0.05, **p < 0.01 vs. NC or NC + Pegmolesatide; #*p* < 0.05 vs. CKD group; ns, no significant difference**).**

To further validate the direct protective effects of Pegmolesatide, an *in vitro* model of cardiomyocyte injury was established using the human AC16 cell line stimulated with serum derived from patients with chronic kidney disease (Figure 6A). A Cell Count Kit-8 (CCK-8) assay was conducted to ascertain the non-cytotoxic concentration range of Pegmolesatide for subsequent experiments. The results demonstrated that at concentrations ranging from 0 to 50,000 ng/mL, Pegmolesatide exhibited no significant effect on cell viability (Figure 6B). RT-PCR analyses confirmed a significant increase in the mRNA levels of key hypertrophic markers, including BNP and ANP, as well as fibrosis-associated genes such as α-smooth muscle actin (α-SMA) and collagen I (Figures 6C–F). Subsequent Western blot analysis revealed that stimulation with CKD serum significantly increased the expression of the hypertrophy marker BNP, which was significantly reduced by Pegmolesatide treatment. However, while a decreasing trend in β-MHC expression was observed following treatment, it did not reach statistical significance (Figures 6G,H). Pegmolesatide treatment effectively counteracted the CKD serum-induced alterations, significantly reducing not only the expression of hypertrophy markers but also transcript levels of key fibrosis-related indicators. The findings from *in vitro* experiments demonstrate that Pegmolesatide exerts direct suppressive effects on CKD serum-induced cardiomyocyte hypertrophy and pro-fibrotic responses.

### 3.6 Pegmolesatide attenuates STAT3 activation and mitochondrial dysfunction in cardiomyocytes

**FIGURE 7:**
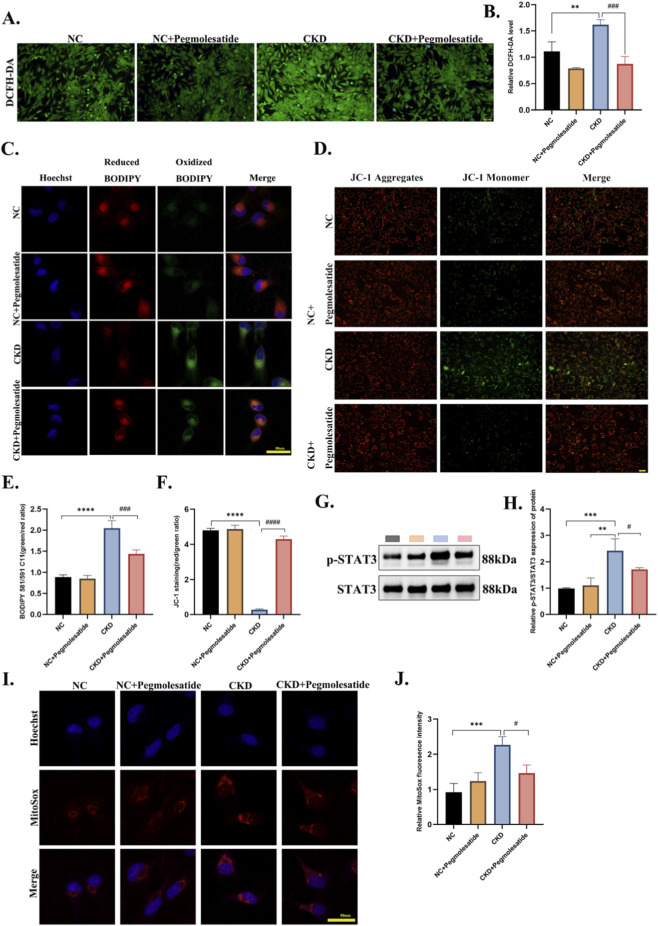
Effects of Pegmolesatide on Oxidative Stress, Mitochondrial Membrane Potential, and STAT3 Phosphorylation in AC16 cells. **(A,B)** ROS levels detected by DCFH-DA staining (green) in 4 cell groups: NC, NC + Pegmolesatide, CKD, and CKD + Pegmolesatide, scale bar = 100 μm. **(C ,E)** Lipid peroxidation levels evaluated by dual-color fluorescence staining (red and green fluorescence); the CKD group showed the weakest red fluorescence intensity accompanied by the strongest green fluorescence intensity. Scale bar = 50 μm. **(D,F)** Mitochondrial membrane potential assessed by JC-1 staining: red indicates JC-1 aggregates (normal potential), green indicates JC-1 monomers (depolarization); Merge shows the overlay, scale bar = 50 μm. **(G,H)** Western blot analysis of p-STAT3 and STAT3 expression. **(I,J)** Mitochondrial superoxide levels detected by MitoSOX Red staining (red fluorescence) in four AC16 cell groups: NC, NC + Pegmolesatide, CKD, and CKD + Pegmolesatide; the CKD group exhibited the most intense red fluorescence. Scale bar = 50 μm (n = 3, **p < 0.01, ***p < 0.001 vs. NC or NC + Pegmolesatide; #*p* < 0.05 vs. CKD group).

To evaluate the protective effects of Pegmolesatide on mitochondrial integrity and function, a series of mitochondrial functional assays were performed in AC16 cardiomyocytes exposed to chronic kidney disease (CKD) serum. Intracellular reactive oxygen species (ROS) levels, measured using the fluorescent probe DCFH-DA, were significantly elevated by CKD serum stimulation, indicating pronounced oxidative stress. Pegmolesatide co-treatment effectively attenuated this increase in a concentration-dependent manner (Figures 7A,B). The assessment of mitochondrial superoxide levels was conducted by means of the MitoSOX™ Red Mitochondrial Superoxide Indicator. CKD serum stimulation led to a significant increase in MitoSOX fluorescence intensity, which was markedly reduced by Pegmolesatide treatment, indicating its specific efficacy in mitigating mitochondrial superoxide production (Figures 7I,J). Assessment of lipid peroxidation using the BDPY581/591C11 fluorescent probe revealed a marked shift from predominant red fluorescence (reduced state) to intense green fluorescence (oxidized state) in the CKD group, indicating substantial lipid oxidative damage. Pegmolesatide treatment led to a significant increase in the red-to-green fluorescence ratio, indicative of reduced lipid peroxidation (Figures 7C,E). The mitochondrial membrane potential (ΔΨm) was assessed using the JC-1 probe. In the context of functional mitochondria, JC-1 adopts a red fluorescent aggregate configuration. Conversely, mitochondrial depolarization is typified by a transition toward green monomeric fluorescence. CKD serum stimulation significantly reduced the red/green fluorescence intensity ratio, indicating a loss of ΔΨm. Pegmolesatide treatment led to a significant preservation of this ratio, suggesting a stabilization of mitochondrial membrane integrity (Figures 7D,F). Furthermore, an examination of STAT3 phosphorylation levels in AC16 cells was conducted. In accordance with the findings derived from experimental observations in animal models, the stimulation of CKD patient serum has been demonstrated to exhibit a significant response (Figures 7G,H).

### 3.7 Pharmacological validation of the JAK2/STAT3 pathway in cardiomyocytes

**FIGURE 8:**
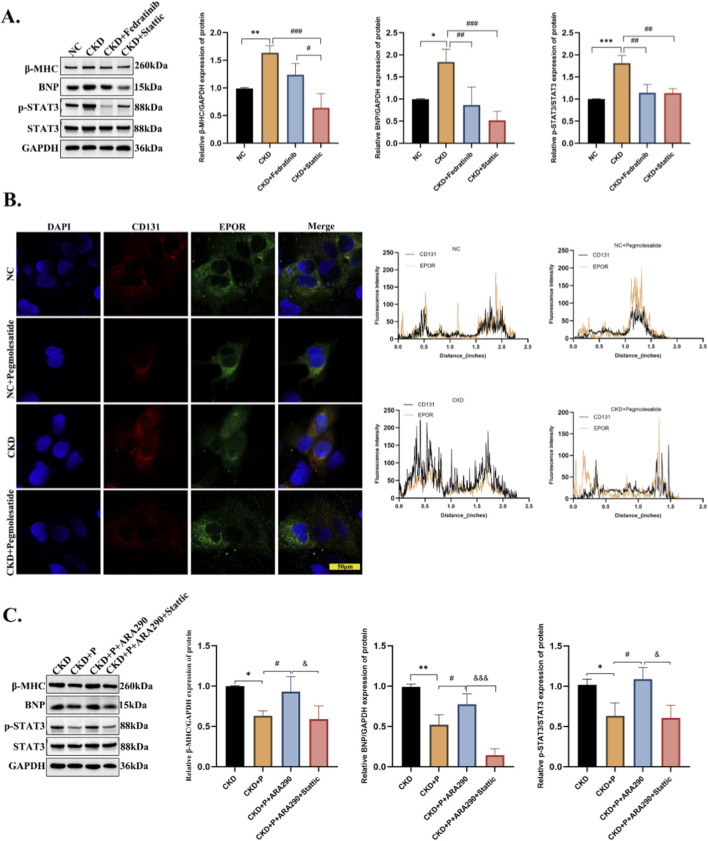
Pharmacological validation of the JAK2/STAT3 pathway and EPOR/CD131 regulation in CKD-induced cardiomyocyte injury. **(A)** Representative Western blots and quantitative analysis of β-MHC, BNP, p-STAT3, and total STAT3 protein levels are shown. GAPDH was used as the loading control. **(B)** Immunofluorescence staining of EPOR (green) and CD131 (red) in AC16 cells, with DAPI (blue) for nuclear counterstaining. Co-localization analysis of EPOR and CD131 was performed, and representative fluorescence intensity profiles are presented. Scale bar, 50 µm. **(C)** AC16 cells were treated with pegmolesatide (P) alone, or in combination with the CD131 agonist ARA290 (10 µM) and/or Stattic 5 µM) under CKD serum stimulation. Representative Western blots and quantitative analysis of β-MHC, BNP, p-STAT3, and total STAT3 are shown. GAPDH was used as the loading control. Data are presented as mean ± SD. *p < 0.05, **p < 0.01, ***p < 0.001 vs. NC group; #*p* < 0.05, ##*p* < 0.01, ###*p* < 0.001 vs. CKD group; & *p* < 0.05, &&& *p* < 0.001 vs. CKD + P group.

To further validate the causal involvement of the JAK2/STAT3 pathway, we performed pharmacological inhibition experiments in AC16 cardiomyocytes stimulated with CKD patient serum. Treatment with the selective JAK2 inhibitor fedratinib (2 µM) or the STAT3 inhibitor Stattic (5 µM) significantly attenuated CKD serum-induced increases in β-MHC and BNP protein levels, and concomitantly suppressed STAT3 phosphorylation (Figure 8A). These data functionally confirm that JAK2 acts upstream of STAT3 in this pathological setting. To examine whether pegmolesatide directly modulates EPOR/CD131 receptor expression, we performed immunofluorescence staining. CKD serum stimulation markedly upregulated CD131 expression and enhanced EPOR/CD131 co-localization in AC16 cells, while pegmolesatide treatment partially reversed these changes (Figure 8B). To further verify the role of CD131 in CKD-induced cardiomyocyte hypertrophy, we conducted pharmacological intervention experiments. Activation of CD131 with its agonist significantly exacerbated CKD-induced cardiomyocyte hypertrophy, as evidenced by elevated β-MHC and BNP protein levels and enhanced STAT3 phosphorylation. Notably, co-treatment with the STAT3 inhibitor Stattic effectively alleviated this CD131 agonist-induced hypertrophic phenotype (Figure 8C).

**FIGURE 9:**
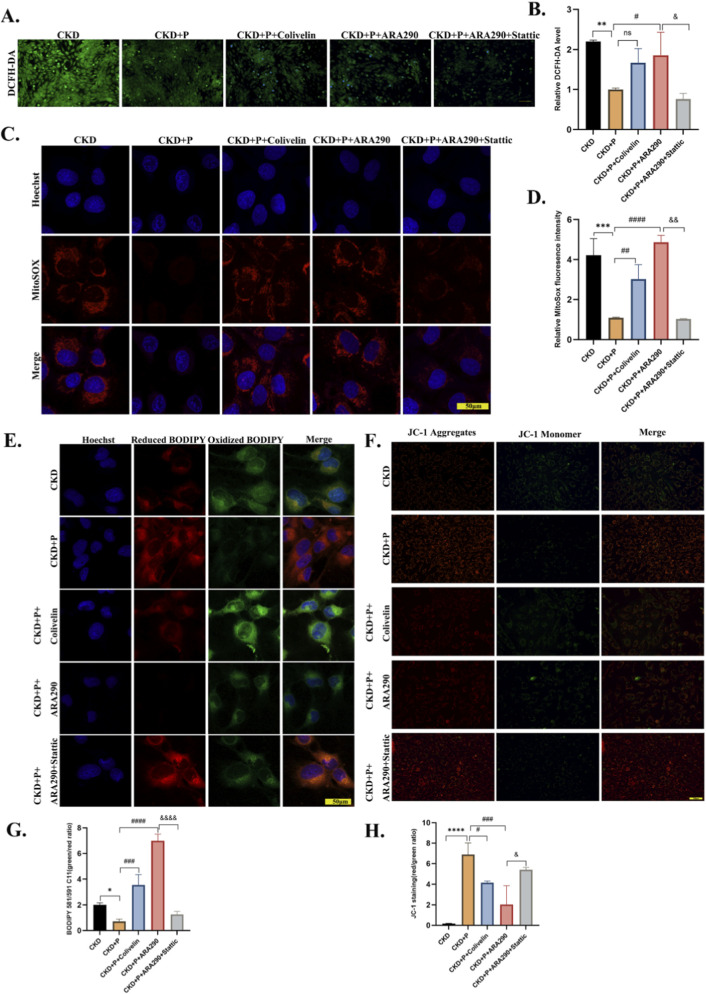
STAT3 inhibition mediates the mitochondrial protective effects of pegmolesatide in CKD-induced cardiomyocyte injury. **(A)** Representative DCFH-DA staining images showing intracellular reactive oxygen species (ROS) levels in AC16 cardiomyocytes under different treatments. **(B)** Quantitative analysis of relative DCFH-DA fluorescence intensity. **(C)** Representative MitoSOX Red staining images showing mitochondrial ROS levels, with Hoechst 33,342 (blue) for nuclear counterstaining. **(D)** Quantitative analysis of relative MitoSOX fluorescence intensity. **(E)** Representative BODIPY 581/591 C11 staining images showing lipid peroxidation levels (oxidized: green, reduced: red), with Hoechst 33,342 (blue) for nuclear counterstaining. **(G)** Quantitative analysis of the ratio of oxidized to reduced BODIPY fluorescence. **(F)** Representative JC-1 staining images showing mitochondrial membrane potential (ΔΨm; aggregates: red, monomer: green). **(H)** Quantitative analysis of the ratio of JC-1 aggregates to monomers. AC16 cells were stimulated with CKD patient serum, and treated with pegmolesatide (P) alone, or in combination with the STAT3 agonist colivelin (1 µM), ARA290 (10 µM), and/or the STAT3 inhibitor Stattic (5 µM). Scale bars, 50 µm.Data are presented as mean ± SD. *p < 0.05, **p < 0.01, ***p < 0.001, ****p < 0.0001 vs. CKD group; #*p* < 0.05, ##*p* < 0.01, ###*p* < 0.001,####*p* < 0.0001vs.CKD + Pgroup; &*p* < 0.05, &&*p* < 0.01, &&&*p* < 0.001,&&&&*p* < 0.0001vs. CKD + P + colivelin/CKD + P + ARA290 group.

To comprehensively assess mitochondrial function, we evaluated multiple key parameters including intracellular reactive oxygen species (ROS), mitochondrial ROS, lipid peroxidation, and mitochondrial membrane potential (ΔΨm). Administration of either the CD131 agonist or the STAT3 agonist colivelin (1 µM) abolished the mitochondrial protective effects of pegmolesatide, as reflected by decreased mitochondrial membrane potential and elevated levels of mitochondrial ROS, lipid peroxidation, and total intracellular ROS. In contrast, co-treatment with the STAT3 inhibitor Stattic partially restored the mitochondrial protective effects of pegmolesatide under these conditions (Figures 9A–H). These results suggest that STAT3 inhibition contributes, at least in part, to the observed mitochondrial protection.

## 4 Discussion

In this study, we investigated the cardioprotective effects of Pegmolesatide in uremic cardiomyopathy through a comprehensive series of *in vivo* and *in vitro* experiments. For the *in vivo* assessment, the 5/6 nephrectomy (5/6 Nx) model was employed. The results demonstrated that treatment with Pegmolesatide significantly attenuated pathological cardiac hypertrophy, improved left ventricular diastolic and systolic function, and suppressed the progression of myocardial fibrosis compared to the vehicle-treated control group. It is noteworthy that while Pegmolesatide elicited a clear cardioprotective phenotype, it did not produce a significant improvement in renal function parameters in the model animals. Concurrent *in vitro* experiments involved the stimulation of AC16 human cardiomyocyte-derived cells with serum from patients with stage 5 chronic kidney disease (CKD 5) or healthy volunteers. The findings indicated that Pegmolesatide treatment led to a significant downregulation in the expression of key hypertrophy markers (ANP, BNP, and β-MHC) and a concurrent reduction in the levels of fibrosis-associated proteins (α-SMA and Col-I). In addition, Pegmolesatide demonstrated a substantial capacity to ameliorate uremic serum-induced mitochondrial dysfunction and oxidative stress in cardiomyocytes. Specifically, it stabilized the mitochondrial membrane potential, reduced the overall generation of reactive oxygen species (ROS), and notably decreased mitochondrial superoxide production, as quantified using the MitoSOX™ Red indicator. Concurrently, the assessment of lipid peroxidation using the BDPY581/591C11 probe demonstrated that Pegmolesatide treatment effectively attenuated the uremic serum-induced shift from reduced (red) to oxidized (green) fluorescence, indicating a reduction in lipid oxidative damage. The integrated findings suggest that the benefits of Pegmolesatide are achieved through the direct attenuation of hypertrophy, fibrosis, and mitochondrial impairment in cardiomyocytes.

The JAK2/STAT3 signaling pathway is a well-established central mediator of pathological cardiac remodeling, driving both myocardial hypertrophy and fibrosis through the transcriptional upregulation of fetal genes and extracellular matrix components (Yang D. et al., 2022; Yang et al., 2025). The present findings demonstrate that Pegmolesatide attenuates cardiac hypertrophy and fibrosis in a manner concomitant with the inhibition of JAK2/STAT3 phosphorylation. These results are strongly supported by extant literature (Zhang et al., 2021). For instance, the natural compound periplocymarin has been shown to alleviate pathological cardiac hypertrophy by specifically inhibiting this same pathway, highlighting a conserved mechanism of action across different therapeutic agents (Fan et al., 2022). This consistency reinforces JAK2/STAT3 as a high-value therapeutic target. However, the role of this pathway is not monolithic and exhibits context-dependent nuances (Guo et al., 2024). Although the existing body of research, including our own, provides unequivocal evidence of the pro-fibrotic and pro-hypertrophic role of this factor in conditions such as uremic and pressure-overload cardiomyopathy, some studies conducted on diabetic models have yielded contradictory results, demonstrating both decreased and increased cardiac STAT3 activity (Liu et al., 2021; Ji et al., 2024; Huo et al., 2025). This discrepancy may be attributed to variations in experimental models, the duration of the disease, or critical cell-specific effects. It is noteworthy that, while cardiomyocyte-specific STAT3 deletion can, in certain instances, exacerbate injury, its activation in cardiac fibroblasts is nearly universally profibrotic (Patel et al., 2020).

In the pathogenesis of pathological cardiac hypertrophy, mitochondrial dysfunction represents a critical driver in the progression from compensatory adaptation to decompensation (Pliouta et al., 2025). This pathological process involves complex molecular mechanisms, including impaired mitochondrial biogenesis, abnormal electron transport chain function, and disrupted fatty acid oxidation (Joles et al., 2018). The vicious cycle between energy deficiency and oxidative stress not only directly compromises cardiomyocyte contractility but also accelerates pathological remodeling by activating multiple apoptotic and necrotic pathways (Deshpande et al., 2025). This study demonstrates that Pegmolesatide effectively attenuates cardiac remodeling in a model of uremic cardiomyopathy through a mechanism involving concurrent suppression of JAK2/STAT3 pathway activation and improvement of mitochondrial function. Specifically, we observed that Pegmolesatide significantly reduced phosphorylation levels of JAK2 and STAT3 while effectively preserving mitochondrial structural and functional integrity. In-depth analysis revealed that stimulation with uremic serum significantly elevated mitochondrial superoxide levels in cardiomyocytes, accompanied by severe lipid peroxidation damage. Pegmolesatide treatment specifically reduced mitochondrial superoxide generation, mitigated lipid peroxidation and stabilized the mitochondrial membrane potential, directly targeting the core aspect of mitochondrial oxidative injury in uremic cardiomyopathy. This finding carries substantial significance as it suggests Pegmolesatide may exert protective effects through a multi-target mechanism operating at both molecular signaling and cellular energy metabolism levels. Viewing mitochondrial dysfunction as a central player in the pathogenesis of uremic cardiomyopathy, the improvement of mitochondrial oxidative stress by Pegmolesatide likely disrupts key downstream pathways leading to cardiomyocyte hypertrophy, fibrosis, and ultimately cell death. Although the precise role of STAT3 in this process, particularly its direct or indirect involvement in mitochondrial regulation, requires further elucidation, the synchronous achievement of JAK2/STAT3 pathway inhibition and mitochondrial functional improvement provides a novel perspective for understanding Pegmolesatide’s multi-faceted protective effects (Jeong et al., 2009; Lu et al., 2025). This dual mechanism of action, simultaneously regulating signal transduction and energy metabolism, represents a coordinated approach against the pathological progression of uremic cardiomyopathy. The distinctive characteristics of this dual mechanism also position Pegmolesatide with unique therapeutic advantages compared to conventional single-target agents. Notably, the cardioprotective effects of Pegmolesatide were observed in a setting of CKD-associated anemia and reduced hemoglobin. The resultant decline in oxygen-carrying capacity can itself induce compensatory hemodynamic changes and alter myocardial metabolism, introducing potential confounding effects on functional and structural assessments. Thus, interpretation of the drug’s direct cardiac mechanisms requires careful distinction from benefits secondary to improved oxygen delivery. Future studies should use controls to separate these direct effects from anemia correction.

While pegmolesatide is well characterized as an erythropoiesis-stimulating agent (ESA) for renal anemia management, our findings reveal novel, previously unrecognized cardioprotective effects of this agent. This PEGylated EPO-mimetic peptide demonstrates enhanced selectivity for the EPOR homodimer, a structural characteristic that underlies its improved hematological efficacy while potentially minimizing pleiotropic effects associated with conventional ESAs (Ma et al., 2025). Notably, our investigation demonstrates that Pegmolesatide directly attenuates cardiac pathology in uremic cardiomyopathy through dual mechanisms: suppression of JAK2/STAT3 signaling activation and preservation of mitochondrial function against uremic toxin-induced damage. The observed divergence between its erythropoietic function—primarily mediated through JAK2/STAT5 activation—and its cardioprotective effects involving JAK2/STAT3 inhibition suggests the engagement of distinct signaling cascades. This mechanistic dichotomy implies the potential involvement of previously unrecognized targets, which may include alternative receptor conformations or tissue-specific signaling complexes modulated by its unique molecular architecture. Further investigation is warranted to delineate the precise molecular interfaces through which Pegmolesatide orchestrates cardiac protection, particularly its regulation of STAT3 signaling networks and mitochondrial bioenergetics, which may reveal novel therapeutic targets for uremic cardiomyopathy beyond conventional anemia management strategies.

From a clinical perspective, the findings of this study suggest that Pegmolesatide may offer a dual benefit in patients with chronic kidney disease (CKD): effective management of renal anemia and direct myocardial protection in the setting of uremic cardiomyopathy. This dual mechanism may prove particularly salient in CKD populations, where cardiac morbidity is prevalent and conventional hemoglobin-target therapies have demonstrated restricted cardiovascular benefit. However, it is imperative to acknowledge several limitations inherent to this approach. Firstly, the mechanistic claim would be strengthened by the use of a CD131 or an EPOR antagonist to confirm receptor-specificity in cardiomyocytes. Secondly, the 5/6 Nx model of uremic cardiomyopathy and the AC16 cell line, though widely utilized, do not fully recapitulate all aspects of human uremic cardiomyopathy, and translation to the human condition remains uncertain. Thirdly, the observed cardioprotection may involve both direct effects and indirect benefits from hemoglobin correction; future studies using a conventional ESA with matched hemoglobin levels are required to confirm anemia-independent action. Fourth, our *in vivo* experiments used only male rats, introducing sex bias. Sex differences affect CKD and cardiovascular disease progression, limiting the generalizability of our findings to female patients. However, our *in vitro* experiments used pooled serum from both male and female CKD stage 5 patients, partially mitigating this concern. Future studies will include both sexes to assess pegmolesatide’s cardioprotective effects in females. Consequently, the execution of extensive clinical trials with cardiovascular endpoints will be imperative to ascertain the cardioprotective efficacy of Pegmolesatide in enhancing outcomes among CKD patients.

## 5 Conclusion

Pegmolesatide significantly attenuates cardiomyopathy progression in a 5/6 nephrectomy rat model of Chronic Kidney Disease. The cardioprotective effects of the drug include reduced myocardial fibrosis, attenuated cardiac hypertrophy, and improved cardiac function. Mechanistically, Pegmolesatide exerts its inhibitory effect on JAK2/STAT3 signaling, with a particular focus on STAT3 phosphorylation. It is noteworthy that the present study is the first to demonstrate that Pegmolesatide directly preserves mitochondrial function by reducing superoxide production and lipid peroxidation, independent of its erythropoietic action. These findings provide preclinical support for the use of the drug in cardiovascular complications related to chronic kidney disease (CKD) and unveil a novel, anemia-independent cardioprotective mechanism.

## Data Availability

The original contributions presented in the study are included in the article/Supplementary Material, further inquiries can be directed to the corresponding author.
