# Supplementary material for: Amelioration of CKD-induced cardiomyocyte hypertrophy by pegmolesatide: involvement of JAK2/STAT3 inhibition and mitochondrial protection
Source: Front Pharmacol. 2026 May 13;17:1806374. doi: 10.3389/fphar.2026.1806374 (PMC13212317; doi:10.3389/fphar.2026.1806374)
Supplement: Supplementary file 1 [file Supplementaryfile1.docx]

| **Parameter** | **Control（n=34）** | **CKD（n=35）** | **P Value** |
| --- | --- | --- | --- |
| Male | 20（58.8%） | 19（54.3%） | 0.891 |
| Age（year） | 41.12±7.66 | 45.43±6.27 | 0.013 |
| Dialysis age（month） | － | 108.89±81.92 | － |
| Hemoglobin（g/L） | 141.35±10.14 | 114.63±8.99 | ＜0.001 |
| SCr（μmol/L） | 77.62±8.74 | 1166.14±230.24 | ＜0.001 |
| BUN（mmol/L） | 5.44±0.57 | 28.22±5.31 | ＜0.001 |
| PROBNP（pg/mL） | － | 4513.54±5964.49 | － |
| β2-microglobulin | － | 36.88±6.93 | － |
| SBP（mmHg） | 119.29±5.35 | 147.01±16.05 | ＜0.001 |
| DBP（mmHg） | 77.06±4.14 | 82.93±11.03 | 0.005 |

**Supplementary Table S1 Baseline characteristics of study participants for in vitro serum stimulation experiments：**Abbreviations: SCr, serum creatinine; BUN, blood urea nitrogen; PROBNP, N-terminal pro-B-type natriuretic peptide; SBP, systolic blood pressure; DBP, diastolic blood pressure.

**Supplementary Figure S1**


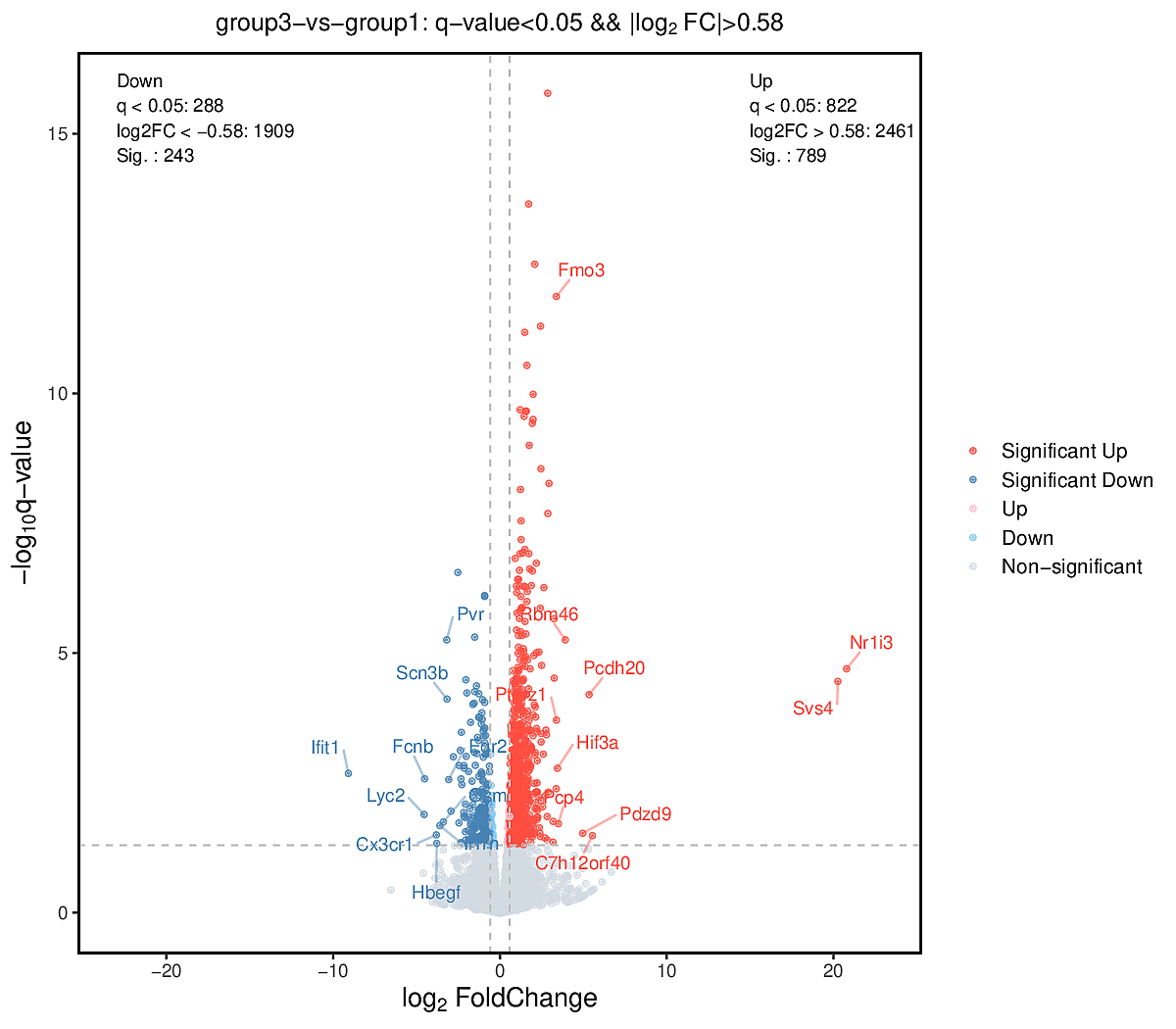


**Volcano plot of DEGs between CKD (group3) and Sham (group1) groups.**

Volcano plot showing differentially expressed genes in rat myocardial tissues. Red: significantly upregulated; blue: significantly downregulated; gray: non-significant. Threshold: q < 0.05 and |log₂FC| > 0.58.

**Supplementary Figure S2**

**
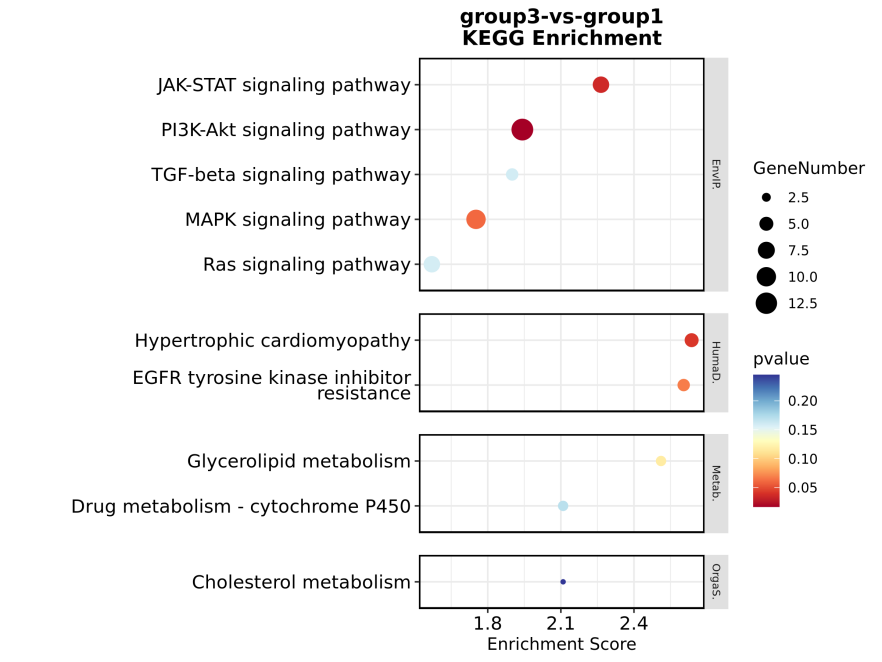
**

**KEGG pathway enrichment analysis of DEGs between CKD (group3) and Sham (group1) groups：**Bubble plot showing the top enriched KEGG pathways in rat myocardial tissues. Enrichment score is shown on the x-axis. Color gradient represents p-value (darker red indicates higher significance), and bubble size represents the number of enriched genes.
